# Editorial: Immune-Related Adverse Events for Patients With Lung Cancer

**DOI:** 10.3389/fonc.2022.898576

**Published:** 2022-05-04

**Authors:** Ruoning Yang, Haiyang Wang, Xiaomeng Yin, Benjamin Frey, Udo S. Gaipl, Hubing Shi, Xuelei Ma

**Affiliations:** ^1^ Department of Biotherapy, State Key Laboratory of Biotherapy, Cancer Center, West China Hospital, Sichuan University, Chengdu, China; ^2^ Department of Breast Surgery, Clinical Research Center for Breast, West China Hospital, Sichuan University, Chengdu, China; ^3^ Department of Otolaryngology Head and Neck Surgery, West China Hospital, Sichuan University, Chengdu, China; ^4^ Translational Radiobiology, Universitätsklinikum Erlangen, Erlangen, Germany

**Keywords:** editorial, lung cancer, immunotherapy, immune-related adverse events (IRAEs), management, pathogenesis

Currently, immune checkpoint inhibitors (ICIs) including programmed cell death-1 (PD-1) inhibitors, programmed cell death ligand 1 (PD-L1) inhibitors, and cytotoxic T-lymphocyte antigen 4 (CTLA-4) inhibitors, show overall improvement of clinical outcomes and better tolerance for patients with lung cancer. However, many immune-related adverse events (irAEs) induced by immunotherapy are reported, which are considered to be a major challenge for immunotherapy. The most common management strategy for irAEs is early prevention, early detection and early treatment. This Research Topic recruited studies that discuss new discoveries in the field of pathogenesis and management of irAEs for patients with lung cancer.

We are very pleased to note that so many excellent work was submitted to these important topics. Finally, 15 papers were published, including two case reports. The research was carried out in different countries, including China, USA and Italy. The papers discuss the occurrence of irAEs, common irAEs, and the management of irAEs. Some studies comprehensively summarized the mechanism, diagnosis, and management of irAEs in patients with lung cancer, including immunotherapy and multimodal therapies (Fu et al., Hou et al., Wang et al., Li et al., Zheng and Wei, and Zhao et al.). Specific irAEs were partly discussed in detail. For example, Zhou and Wei reviewed immune checkpoint inhibitor (ICI) associated ocular side effects in lung cancer, and Zhang et al. and Zhu et al. both discussed another important irAE, namely checkpoint inhibitor-induced pneumonitis (CIP). And Tian et al. conducted a systematic review and meta-analysis to reveal the relationship between PD-1/PD-L1 inhibitors and neurological toxicities among cancer patients. As it is known to us, irAEs can happen to any organ, such as lung, liver, skin, kidney, digestive system, or endocrine system. Simultaneous involvement of multiple organs is rare but still reported. Deng et al. reported about a 71-year-old man with NSCLC showing severe multiple-organs injuries after tislelizumab treatment, which provides a reference for the management of multiple-organs irAEs. Proper management is important to mitigate the negative effects of irAEs. Most of irAEs are mild and can be managed through transient immunosuppression with corticosteroids. Thus, immunotherapy can continue under close monitoring after mild irAEs. The incidence of moderate to severe irAEs is very low, but it can lead to serious organ dysfunction and even death. Sometimes, discontinuing current therapy is necessary. Studies about the clinical outcomes of patients with lung cancer following immunotherapy interruption because of irAEs are still scarce, and there is no consensus on whether treatment interruption will affect disease progression. Damato et al. reported about a woman with NSCLC that discontinued pembrolizumab because of severe colitis, keeping a partial response of the oncological disease in the following 24 months, but not completely recovering from colitis. This case showed that the treatment interruption didn’t compromise the control of the oncological disease. The management of lung cancer patients with a history of prior autoimmune disease (AID) is another controversial topic. It has recently been reported that irAEs are more common among patients with autoimmune diseases and previous viral infections, which are patients with preexisting antibodies (Zheng and Wei). Tang et al. discussed the efficacy and safety of ICIs in patients with cancers and AID. They proposed that although irAEs occur more frequently, AID isn’t an absolute contraindication for ICI treatment. Patients with AID need more close administration to reduce the injury of irAEs.

Compared with other irAEs, CIP is more worthy of our concern and vigilance. For patients with lung cancer, immune-mediated lung injury occurs in about 3% to 5% of patients receiving immunotherapy, which is higher than that in patients with other cancers (1). And the symptoms of CIP will overlap with the original respiratory signs, which makes diagnosis very difficult. CIP is usually an exclusionary diagnosis, and particularly, CIP may appear several months after the end of treatment. Accurate diagnosis is of primary importance, especially the level assessment. Corticosteroid regimen for CIP is still being explored (Zhang et al.). As for the risk factors for lung cancer, there is no consensus. For clinicians, they should focus on patients with a history of smoking, previous radiation therapy and previous lung disease (Zhu et al.).

In recent years, the prospect of adequate biomarkers in immunotherapy has gradually emerged. By having biomarkers for prognosis and prediction, we aim to achieve tailored treatment for each patient, resulting in maximizing the probability of response while minimizing the occurrence of irAEs, and thus reducing the harm of treatment. Unfortunately, till now, there have been no useful predictive biomarkers to assess the development of immune-related adverse events in the clinic (Burke and Rashdan). Currently, no validated biomarkers to predict irAEs induced by ICIs, not only for lung cancer but for all solid tumors, are available. Although CD8+ T cells and Interleukin 17 were reported to have connections with irAEs, the threshold is still unclear (2). In addition, Zhao et al. proposed that the occurrence of irAEs is strongly associated with better survival and response in NSCLC patients treated with PD-1 inhibitors, suggesting that irAE may be a potential predictive biomarker in this scenario (Zhang et al.).

In conclusion, there are many studies on irAEs in patients with lung cancer, but the diagnosis and management of irAEs still require more exploration. Firstly, timely and accurate diagnosis is essential. Secondly, risk stratification of irAEs is one basis of treatment. Thirdly, until today there is no optimal strategy for the pharmacotherapy of irAEs, which requires more time and larger clinical sample size to be evaluated. More and more excellent research will contribute to this field in the future.

This Research Topic accepted many excellent studies, mainly involving the diagnosis, grading and management of adverse events. We hope to improve the quality of life of patients with lung cancer through a better understanding of irAEs. We appreciate all the reviewers and authors for their contributions to this Research Topic. We hope this Research Topic can even arise more attention in the related fields.

## Author Contributions

Literature review, and data collection and were performed by RJ, XY and HW. The first draft of the manuscript was written by RY and XM. The final version of the editorial was written by USG, BF and HS. All authors read and approved the final manuscript.

## Conflict of Interest

The authors declare that the research was conducted in the absence of any commercial or financial relationships that could be construed as a potential conflict of interest.

## Publisher’s Note

All claims expressed in this article are solely those of the authors and do not necessarily represent those of their affiliated organizations, or those of the publisher, the editors and the reviewers. Any product that may be evaluated in this article, or claim that may be made by its manufacturer, is not guaranteed or endorsed by the publisher.
